# Surveillance for Hospitalized Acute Respiratory Infection in Guatemala

**DOI:** 10.1371/journal.pone.0083600

**Published:** 2013-12-31

**Authors:** Jennifer R. Verani, John McCracken, Wences Arvelo, Alejandra Estevez, Maria Renee Lopez, Lissette Reyes, Juan Carlos Moir, Chris Bernart, Fabiola Moscoso, Jennifer Gray, Sonja J. Olsen, Kim A. Lindblade

**Affiliations:** 1 Respiratory Diseases Branch, Centers for Disease Control and Prevention, Atlanta, Georgia, United States of America; 2 Centro de Estudios en Salud, Universidad del Valle, Guatemala City, Guatemala; 3 International Emerging Infections Program, Centers for Disease Control and Prevention, Regional Office for Central America and Panama, Guatemala City, Guatemala; 4 Division of Global Disease Detection and Emergency Response, Centers for Disease Control and Prevention, Atlanta, Georgia, United States of America; 5 Field Epidemiology Training Program, Ministerio de Salud Pública y Asistencia Social, Guatemala City, Guatemala; 6 Área de Salud de Santa Rosa, Ministerio de Salud Pública y Asistencia Social, Cuilapa, Guatemala; 7 Área de Salud de Quetzaltenango, Ministerio de Salud Pública y Asistencia Social, Quetzaltenango, Guatemala; 8 Influenza Division, Centers for Disease Control and Prevention, Atlanta, Georgia, United States of America; Naval Medical Research Unit 6, United States of America

## Abstract

Acute respiratory infections (ARI) are an important cause of illness and death worldwide, yet data on the etiology of ARI and the population-level burden in developing countries are limited. Surveillance for ARI was conducted at two hospitals in Guatemala. Patients admitted with at least one sign of acute infection and one sign or symptom of respiratory illness met the criteria for a case of hospitalized ARI. Nasopharyngeal/oropharyngeal swabs were collected and tested by polymerase chain reaction for adenovirus, parainfluenza virus types 1,2 and 3, respiratory syncytial virus, influenza A and B viruses, human metapneumovirus, *Chlamydia pneumioniae*, and *Mycoplasma pneumoniae*. Urine specimens were tested for *Streptococcus pneumoniae* antigen. Blood culture and chest radiograph were done at the discretion of the treating physician. Between November 2007 and December 2011, 3,964 case-patients were enrolled. While cases occurred among all age groups, 2,396 (60.4%) cases occurred in children <5 years old and 463 (11.7%) among adults ≥65 years old. Viruses were found in 52.6% of all case-patients and 71.8% of those aged <1 year old; the most frequently detected was respiratory syncytial virus, affecting 26.4% of case-patients. Urine antigen testing for *Streptococcus pneumoniae* performed for case-patients ≥15 years old was positive in 15.1% of those tested. Among 2,364 (59.6%) of case-patients with a radiograph, 907 (40.0%) had findings suggestive of bacterial pneumonia. Overall, 230 (5.9%) case-patients died during the hospitalization. Using population denominators, the observed hospitalized ARI incidence was 128 cases per 100,000, with the highest rates seen among children <1 year old (1,703 per 100,000), followed by adults ≥65 years old (292 per 100,000). These data, which demonstrate a substantial burden of hospitalized ARI in Guatemala due to a variety of pathogens, can help guide public health policies aimed at reducing the burden of illness and death due to respiratory infections.

## Introduction

Acute respiratory infections (ARI) are a leading killer of children worldwide and also cause substantial morbidity and mortality among adults [1], [2]. ARI disproportionately affects the world's poorest populations [1], [3]. However, ascertaining the true burden of ARI in developing countries is challenging. Clinical case definitions are variable and non-specific, and laboratory and radiology diagnostics that can improve the accuracy of ARI surveillance are not always available. Active, population-based surveillance for ARI cases in the community is extremely resource-intensive and difficult to carry out for large populations. On the other hand, health facility-based surveillance is affected by healthcare seeking patterns for ARI, which may vary by population group and over time [4].

The etiology of ARI in developing countries is also not well characterized. Diagnostic testing for the broad range of pathogens that can cause ARI is costly and not widely available in resource-poor settings. Even with optimal diagnostic testing, the etiology of ARI can be difficult to determine due to limitations in available diagnostic methods. Furthermore, the causes of ARI are dynamic, with some pathogens emerging or newly recognized (such as influenza A (H1N1)pmd09 virus or human metapneumovirus), and others potentially declining. Vaccines against respiratory pathogens, such as *Haemophilus influenzae* type B or influenza viruses, may impact the incidence of hospitalized ARI as well as the relative contributions of other pathogens to the burden of ARI. Changes in the prevalence of risk factors for ARI, such as crowded living conditions or malnutrition, may also alter the burden of hospitalized ARI over time.

The International Emerging Infections Program of the U.S. Centers for Disease Control and Prevention (CDC), in collaboration with the Guatemala Ministry of Public Health and Welfare and the Universidad del Valle de Guatemala (UVG) conducts surveillance for hospitalized ARI in two sites in Guatemala. The surveillance is aimed at measuring the burden of hospitalized ARI in the catchment area and characterizing ARI etiology. We present the findings of surveillance for hospitalized ARI from November 2007 through December 2011.

## Methods

### Ethics Statement

The surveillance protocol received approval from the institutional review boards of UVG (Guatemala City, Guatemala) and CDC (Atlanta, GA, USA), and approval from the Guatemala Ministry of Public Health and Welfare. Verbal consent was requested of patients in order to screen them for eligibility. Written, informed consent was obtained from eligible patients willing to participate. For patients <18 years of age, parents or guardians were asked to provide written, informed consent for the participation of the patient, and children aged seven through 17 years were asked for written, informed assent.

### Setting

The surveillance system for hospitalized ARI is part of an on-going, integrated, health facility-based surveillance for respiratory, diarrheal, neurologic and febrile illness carried out in two departments – Santa Rosa (total population 319, 963), located 50 km south-east of the capital, Guatemala City, and Quetzaltenango (total population 705, 301), located 120 km north-west of the capital. Surveillance is conducted at the primary public hospital in each of the departments and both facilities serve as the regional reference hospitals. In Santa Rosa, surveillance of hospitalized ARI began in November 2007, and is conducted at the National Hospital of Cuilapa, which is the only public hospital in the department; it is a government hospital with 176-bed capacity, including four pediatric intensive care unit (ICU) beds and eight adult ICU beds. In Quetzaltenango, surveillance began in February 2009, and is conducted at the Western Regional Hospital, one of two general hospitals in the department; it is a large government hospital with 425 beds, including 22 pediatric and six adult ICU beds.

Within each department, a surveillance catchment area was defined based on the municipalities of residence of people visiting the emergency department. Healthcare utilization surveys were carried out at each site to characterize patterns of accessing healthcare for the illnesses under surveillance. In Santa Rosa, the survey was carried out in 2006 and found that among people who were hospitalized for severe respiratory illness (defined as cough and difficulty breathing for ≥2 days and/or report of a diagnosis of pneumonia by a healthcare provider during the last 12 months), 33% of those aged <5 years and 75% of those aged ≥5 years were admitted to the National Hospital of Cuilapa. In Quetzaltenango, the survey was carried out in 2009 and found that 75% of those aged <5 years and 50% of those aged ≥5 years hospitalized with severe respiratory illness were admitted to the Western Regional Hospital [5], [6].

The *Haemophilus influenzae* type B vaccine was introduced into the Guatemalan routine infant immunization program in 2005 [7]. The pneumococcal conjugate vaccine is available on the private market but was not introduced into the routine infant immunization program during the study period. Since 2007, seasonal influenza virus vaccine has been recommended for persons aged 60 and older and health care workers [8], although coverage is low [9].

### Case definition and identification

A case of ARI was defined as a person admitted to one of the surveillance hospitals with at least one sign of acute infection and one sign or symptom of respiratory illness (Table 1). In the hospitals at each site, study nurses reviewed ward registers for patients admitted for respiratory-related diagnoses as well as emergency department logs for patients presenting with respiratory complaints. After obtaining verbal consent, patients admitted with a respiratory-related admission diagnosis or chief complaint were screened for eligibility as ARI cases (Table 1). In addition, patients that were consented and enrolled in the surveillance for acute diarrheal, neurological illness, or fever of unknown etiology were also screened for eligibility as ARI cases.

**Table 1:** Case definition for hospitalized acute respiratory infection*.

Signs of acute infection	Signs or symptoms of respiratory disease
Fever (≥38°C)	Tachypnea
Hypothermia (<35°C)	Cough
Abnormal white blood cell count	Sputum production
<5 years: <5500×10^3^/µL or >15000×10^3^/µL	Pleuritic chest pain
≥5 years: <3000×10^3^/µL or >11000×10^3^/µL	Hemoptysis
Abnormal white blood cell differential*	Difficulty breathing
	Shortness of breath
	Sore throat
	For children <2 years
	Not eating, drinking or breastfeeding
	Pausing repeatedly while drinking or breastfeeding
	Chest indrawing
	Nasal flaring
	Noisy breathing

Any white blood cell differential abnormality as defined by the automated blood cell analyzer at each surveillance site. For Santa Rosa: lymphocytes <25% or >45%, monocytes <2% or >10%, granulocytes <50% or >70%. For Quetzaltenango: lymphocytes <20% or >50%, monocytes <4% or >8%, neutrophils <40% or >70%, eosinophils >6%, basophils >2%.

### Data and sample collection

Surveys were administered to participants and/or parents/guardians to gather demographic and epidemiologic data and information related to their illness. Additional clinical data, including presenting signs and symptoms, history of chronic illness, vital signs, hematology testing results, clinical course and admission/discharge diagnoses were gathered through medical record abstraction. A study physician performed a respiratory physical examination on all patients who met the case definition. When feasible, study nurses measured peripheral oxygen saturation using a pulse oximeter with the patient off oxygen. Study nurses also took nasopharyngeal (NP) and oropharyngeal (OP) swabs. Urine samples were gathered for enrolled patients ≥15 years of age. Blood cultures were performed per routine clinical care using automated blood culture systems (generally one blood culture bottle for children and two aerobic +/− two anaerobic bottles for adults); results of growth from any bottle were followed and recorded by study nurses. Follow-up contact of enrolled patients was attempted within three to six weeks after discharge to assess post-discharge sequelae or death.

### Standardized interpretation of radiographs

Chest radiographs (CXRs) were performed when indicated as part of routine clinical care; surveillance staff obtained a digital image of CXRs done on enrolled patients using a digital camera [10]–[12]. The digital images were reviewed by a panel of radiologists who had undergone training on the World Health Organization (WHO) guidelines for standardized interpretation of CXRs for the diagnosis of pneumonia in children [12]. A modified version of the guidelines was used to interpret adult CXRs, which included recording the same radiologic endpoints as are used for pediatric CXRs. All digital images were reviewed independently by two radiologists and were classified as having end-point consolidation, other consolidation/infiltrate, no consolidation/infiltrate/effusion or uninterpretable. In cases of discordant interpretations between the first two readers, a third trained radiologist served as arbiter. End-point consolidation was considered suggestive of a bacterial etiology [12].

### Laboratory testing of NP/OP swabs and urine

NP and OP swabs from each patient were placed in one tube in viral transport media that was stored at 4°C and sent to the International Emerging Infections Program laboratory at UVG where they were tested using real-time reverse transcriptase polymerase chain reaction per standard CDC protocols for adenovirus, parainfluenza virus types 1,2 and 3, respiratory syncytial virus (RSV), influenza A and B viruses, human metapneumovirus, *Chlamydia pneumioniae*, and *Mycoplasma pneumoniae* [9], [13], [14]. Samples were processed within 72 hours of being collected. Urine specimens were tested for *Streptococcus pneumoniae* and *Legionella pneumophila* (serogroup 1) antigen using Binax NOW (Binax Inc., Scarborough, ME, USA) tests.

### Data management and analysis

Data collected through questionnaires and medical chart reviews were entered into hand-held personal digital devices with pre-programmed range and logic checks and skip patterns. Unique identifiers were assigned and used to link laboratory, clinical, and epidemiologic data. Data were managed and stored using Microsoft SQL Server 2008 (Redmond, VA, USA) and were imported into SAS Enterprise Guide (Cary, NC, USA) for analysis. OpenEpi version 3.01 [15] was used to calculate 95% confidence intervals (CI) for observed incidence rates.

### Incidence calculations

Cases in patients from the defined catchment areas within each of the departments were used to calculate the incidence of hospitalized ARI. Denominators were the age-specific total populations of the municipalities in the surveillance catchment area obtained from the 2002 national census adjusted for population growth [16]. In addition, we calculated an adjusted incidence of hospitalized ARI, taking into account the findings of the healthcare utilization surveys in each catchment area described above [5], [6]. Observed incidence rates were divided by the following proportions to account for hospitalized ARI cases estimated to have been missed by the surveillance system: Santa Rosa <5 years of age: 0.33; Santa Rosa ≥5 years of age: 0.75; Quetzaltenango <5 years of age: 0.75; Quetzaltenango ≥5 years of age: 0.50. CIs were not calculated for adjusted rates because multiple levels of uncertainty around the estimates limited our ability to precisely quantify the interval. The adjustments were used to estimate hospitalized ARI only, and did not take into account the proportions of people with severe respiratory illness that were not hospitalized or that did not seek care. Data from 2007 were not included in incidence calculations since limited data were available for that year, and the calculations for Quetzaltenango were adjusted for an 11-month period of surveillance in 2009. Case-patients residing outside the catchment area were enrolled but not included in incidence calculations.

## Results

### Hospitalized ARI case-patient characteristics

From November 1, 2007, through December 31, 2011, a total of 8,914 hospitalized patients were screened for possible inclusion at the two hospitals. Among all screened patients, 4,837 (54.3%) presented with a chief complaint of some type of respiratory illness; of those 3,947 (81.6%) met the case definition for hospitalized ARI. An additional 329 hospitalized ARI cases were identified among patients presenting with a non-respiratory complaint, yielding a total of 4,276 patients who met the case definition, of which 3,964 (92.7%) were enrolled. Of those, 2,711 (68.4%) resided in the defined catchment areas in which healthcare utilization surveys had been conducted.

The median age among all enrolled patients was 1.7 years, while the mean age was 18.6 years; the range was 1 day to 100 years. Additional demographic and clinical data are presented in Table 2. There was a slight predominance of males (54.1%) and a majority of patients (63.2%) resided in households where the average monthly income was less than 1,000 Quetzals, (USD ∼$130). Cough, reported in 92.8% of cases, was the most common symptom, followed by difficulty breathing (84.4%). Reported fever (72.4%) was more common than measured fever (43.4%). Of note, antipyretic use was common –61.5% of patients reported taking some medication within the 72 hours preceding admission, and 69.5% of those reported using antipyretics. Antimicrobial use was reported among or 37.4% of all case-patients. The most common physical finding was an abnormal lung exam, including rales, rhonchi or crackles on auscultation (90.3%); wheezing was less common (56.5%).

**Table 2:** Characteristics of patients hospitalized with acute respiratory infection, N = 3,964.

	n (%)
*Patient characteristics*	
Age group	
<1 year	1588 (40.1)
1–4 years	808 (20.4)
5–14 years	294 (7.4)
15–49 years	514 (13.0)
50–64 years	297 (7.5)
65+ years	463 (11.7)
Male	2146 (54.1)
Amerindian indigenous	1386 (35.5)
Monthly income <1,000 Quetzals (∼USD130)	2468 (63.2)
*Signs, symptoms, and physical exam findings*	
Cough	3605 (92.8)
Difficulty breathing	3274 (84.4)
Tachypnea*	2131 (53.8)
Reported fever	2856 (72.4)
Measured temperature ≥38°C	1702 (43.4)
Wheezing on lung exam	2160 (56.5)
Rales, crackles or rhonchi on lung exam	3580 (90.3)
Oxygen saturation measured off oxygen	3266 (82.4)
Hypoxic†	1106 (33.9)
*Clinical course and outcome*	
Sought care prior to hospitalization	2118 (57.4)
Used medication within prior 72 hours	2390 (61.5)
Antipyretics	1635 (69.5)
Antibiotics	1482 (66.3)
Antivirals	31 (1.4)
Chronic medical illness	757 (20.1)
Duration of hospitalization (days)	
Range	1–295
Median	5.5
Intensive care	821 (21.6)
Mechanical ventilation	352 (9.3)
Death (in hospital)	230 (5.9)
Post-discharge follow-up completed	2136 (58.6)
Death (within 7 days of discharge) ‡	58 (2.7)

<2 months: ≥ 60 per minute; 2–12 months:≥50 per minute; >12 months-5 years: ≥40 per minute; >5 years≥20 per minute.

Oxygen saturation <90% in Santa Rosa and <88% in Quetzaltenango.

Follow up data available for 2,136 (58.6%) of 3,734 case-patients discharged alive.

Most patients (57.4%) had sought some care prior to hospitalization; the most common sites reported for initially seeking care were health centers (n = 553, 33.2%) and private clinics (n = 541, 32.5%). The duration of hospitalization ranged from 1 to 295 days, with a median of 5.5 days, and interquartile range of 3.3 to 8.8 days. Illness was severe enough to warrant an admission to the ICU for 21.6% of patients and 9.3% required mechanical ventilation. By age group, the proportion admitted to the ICU were: <1 year: 28.0%; 1–4 years: 24.6%; 5–14 years: 20.1%; 15–49 years: 13.4%; 50–64 years: 10.4%; ≥65 years: 11.1%. The overall in-hospital case fatality proportion was 5.9%; among 2,136 (58.6%) patients with post-discharge follow up information, an additional 58 (2.7%) of patients died within seven days of discharge. By age group, the in-hospital case fatality proportions were: <1 year: 4.8%; 1–4 years: 2.8%; 5–14 years: 2.7%; 15–49 years: 10.4%; 50–64 years: 8.7%; ≥65 years 10.4%.

### Diagnostic testing: viruses

**Figure 1:**
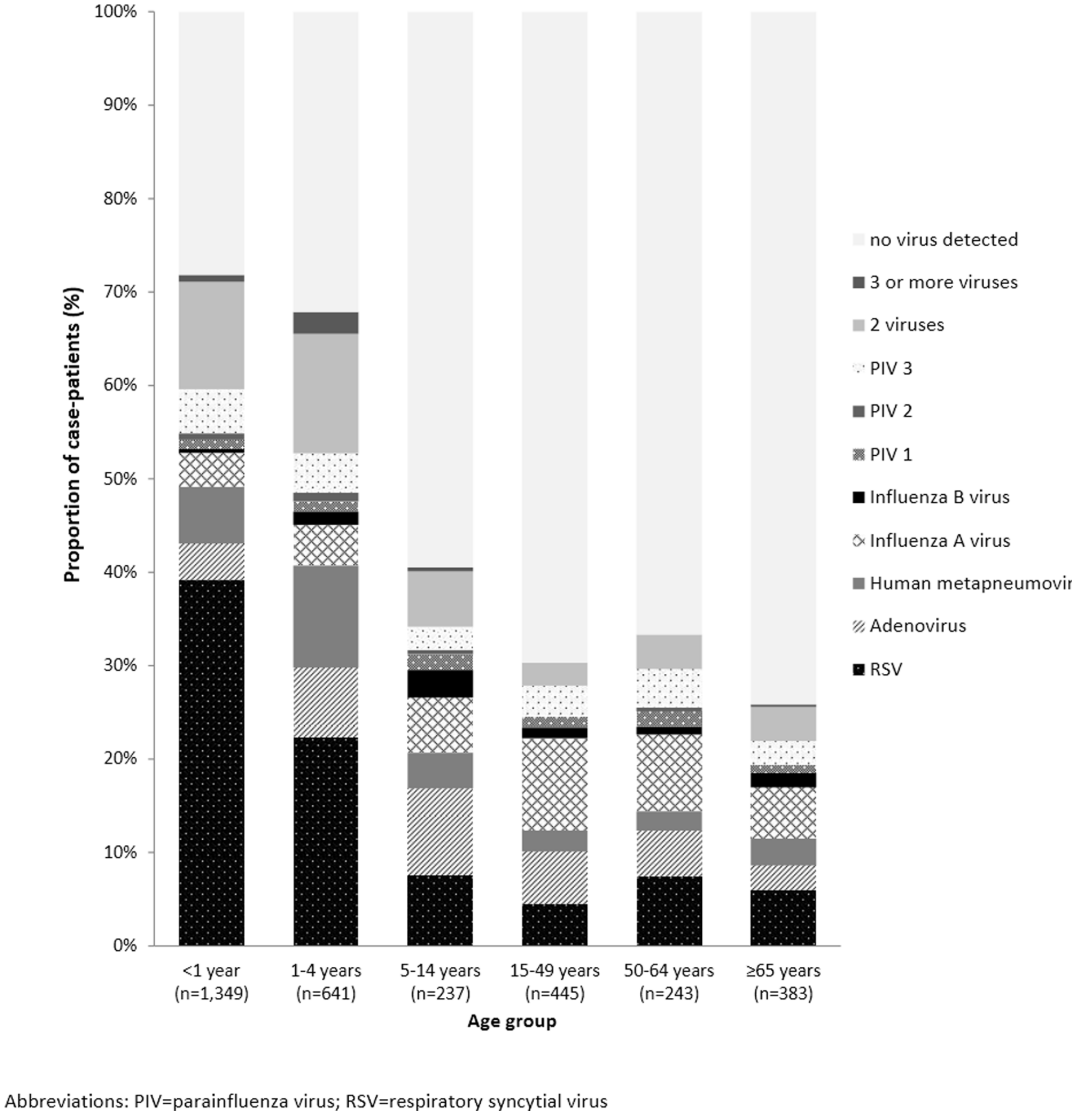
Viral pathogens by age group. Proportion of case-patients with viruses detected on nasopharyngeal/oropharyngeal swab, by age group.

Just over half (50.4%) of case-patients had at least one virus detected, and 365 (9.4%) tested positive for two or more viruses (Table 3). The most commonly detected virus was RSV, affecting 26.4% of all case-patients. The relative frequency of viral pathogens detected in different age groups is presented in Figure 1. Viral infection was more common among case-patients <5 years old compared with those ≥5 years old (69.0% versus 30.3%, p<0.005), yet viruses were detected in all age groups. Infections with RSV and parainfluenza virus type 2 were also most common among children <5 years old, while influenza A virus was slightly more prevalent among older age groups. Multiviral infections were most commonly found among younger age groups. Among 365 case-patients with more than one virus, the most common combination was RSV and adenovirus (n = 84, 23.0%), followed by RSV and parainfluenza virus type 3 (n = 37, 10.1%). RSV was detected in 221 (60.6%) case-patients with more than one virus.

**Table 3:** Diagnostic tests performed, results, and probable etiologies among hospitalized patients with acute respiratory infections, N = 3,964.

	n (%)
*Diagnostic tests*	
Hematology performed	3770 (95.1)
Abnormal WBC*	1610 (42.7)
Abnormal differential†	3502 (94.2)
Nasopharyngeal/oropharyngeal viral testing performed	3883 (98.0)
Respiratory syncytial virus	1024 (26.4)
Adenovirus	434 (11.2)
Human metapneumovirus	300 (7.7)
Influenza A virus	272 (7.0)
Influenza B virus	63 (1.6)
Parainfluenza virus type 1	89 (2.3)
Parainfluenza virus type 2	49 (1.3)
Parainfluenza virus type 3	279 (7.0)
1 virus detected	1722 (44.3)
2 viruses detected	322 (8.3)
>2 viruses detected	43 (1.1)
Nasopharyngeal/oropharyngeal bacterial testing performed	3102 (78.3)
*Chlamydia pneumoniae*	17 (0.6)
*Mycoplasma pneumoniae*	22 (0.7)
Urine antigen for *Streptococcus pneumoniae* performed	1069 (27.0)
*S. pneumoniae*	161 (15.1)
Urine antigen for *Legionella pneumophila* performed	647 (16.3)
*L. pnuemophila (serogroup 1)*	1 (0.2)
Blood culture performed	1443 (36.4)
Results available	1335 (92.5)
No growth	884 (66.2)
*Staphylococcus aureus*	32 (2.4)
*Streptococcus pneumoniae*	12 (0.9)
*Streptococcus* spp.	7 (0.5)
*Escherichia coli*	10 (0.8)
*Klebsiella pneumoniae*	7 (0.5)
* Salmonella* Typhi	5 (0.4)
*Pseudomonas aeruginosa*	3 (0.2)
Coagulase-negative Staphylococci‡	332 (24.9)
Chest radiograph reviewed by radiologist panel§	2364 (59.6)
Image sufficient quality for interpretation	2265 (95.8)
End-point consolidation	907 (40.0)

WBC<5,500 or >15,000×10^3^/µL for <5 years; WBC<3,000 or >11,000×10^3^/µL for ≥5 years.

Any white blood cell differential abnormality as defined by the automated blood cell analyzer at each surveillance site. For Santa Rosa: lymphocytes <25% or >45%, monocytes <2% or >10%, granulocytes <50% or >70%. For Quetzaltenango: lymphocytes <20% or >50%, monocytes <4% or >8%, neutrophils <40% or >70%, eosinophils >6%, basophils >2%.

Excludes 7 case patients with blood cultures positive for coagulase-negative staphylococcus who also had at least one additional blood culture that grew a bacterial respiratory pathogen.

Data were not available for all chest radiographs performed on case-patients.

### Diagnostic testing: bacteria

**Figure 2:**
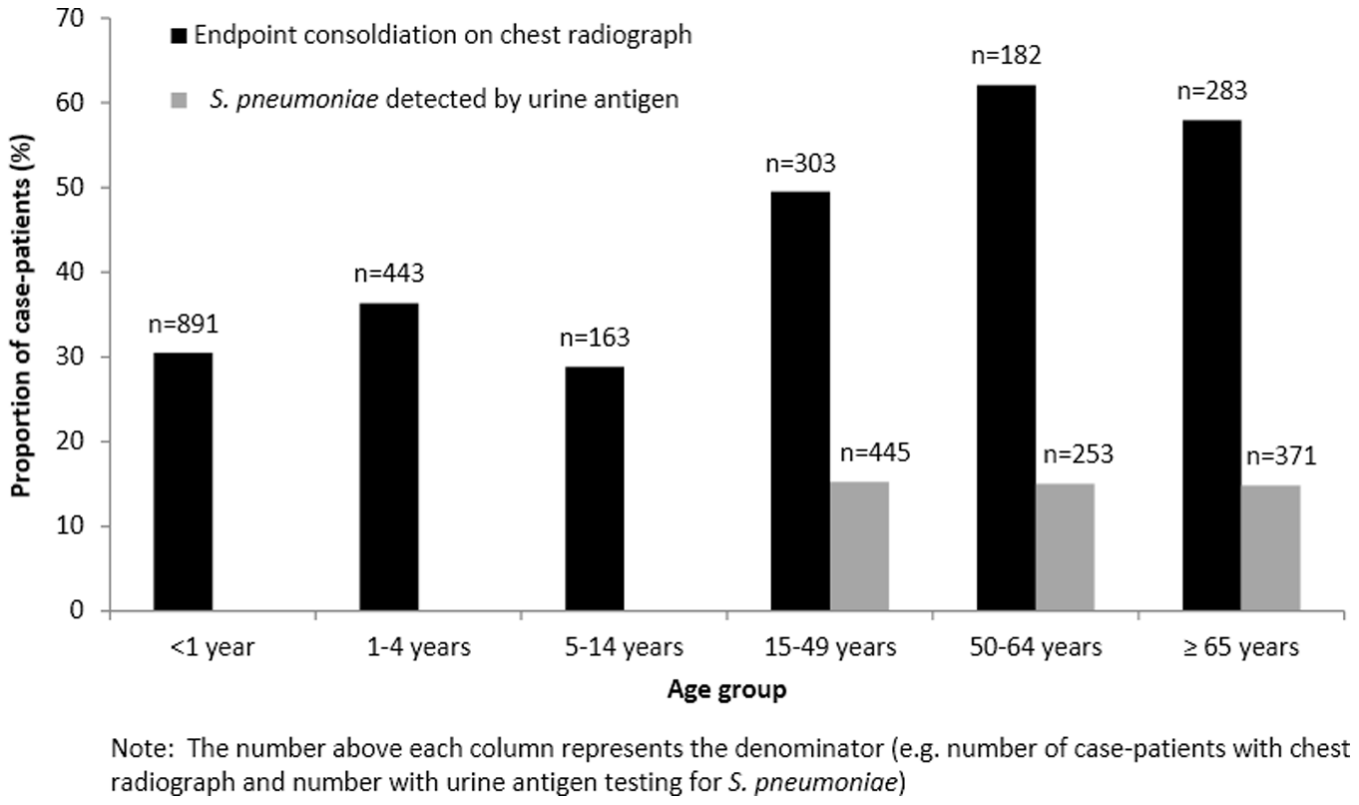
*Streptococcus pneumoniae* and endpoint consolidation by age group. Percent of case-patients with endpoint consolidation on chest radiograph and *Streptococcus pneumoniae* detected by urine antigen, by age group.

Results of bacterial testing of NP/OP swabs, urine and blood culture are presented in Table 3. C. *pneumoniae* and *M. pneumoniae* were rarely detected. Urine antigen testing among those ≥15 years old was positive for *S. pneumoniae* in 15.1% of cases and positive for *L. pneumophila* in only one case. The proportion of adult case-patients with *S. pneumoniae* detected by urine antigen was similar across age groups (Figure 2). Blood cultures were performed for 36.4% of patients and were frequently contaminated, with 24.9% of blood cultures with available results growing only coagulase-negative staphylococci. The most common pathogens isolated among the patients with blood cultures results were *Staphylococcus aureus* (n = 32, 2.4%) and *S. pneumoniae* (n = 12, 0.9%). Among the 12 with *S. pneumoniae* isolated from blood, four were also positive by urine antigen testing, one had a negative urine antigen result, and seven did not have urine antigen testing performed (including two case-patients aged <15 years and five case-patients who were ≥15 years but not tested).

Digital images of CXRs were available for interpretation for 2,364 (59.6%) patients, and 2,265 (95.8%) of those images were considered to be of adequate quality for standardized interpretation. Among interpretable images, 907 (40.0%) were found to show end-point consolidation suggestive of bacterial etiology. As shown in Figure 2, end-point consolidation on CXR was more common among adult age groups. End-point consolidation was found in 32.5% of case-patients <5 years old versus 50.9% of those ≥5 years old (p<0.005).

### Mixed bacterial and viral infections

Among 168 case-patients with *S. pneumoniae* infection (detected by either blood culture or urine antigen testing) and viral testing performed, 48 (28.6%) also had at least one virus detected on NP/OP swab; the most frequently detected virus in this group was influenza A virus (n = 15). Viruses were detected in 22 (68.8%) of the 32 case-patients with *S. aureus* isolated by blood culture; RSV (n = 12) was the virus most commonly found. Among 893 case-patients with end-point pneumonia on CXR and viral testing performed on NP/OP swabs, 437 (48.9%) tested positive for at least one virus, with RSV detected in 176 (19.7%).

### Seasonality and burden of hospitalized ARI

**Figure 3:**
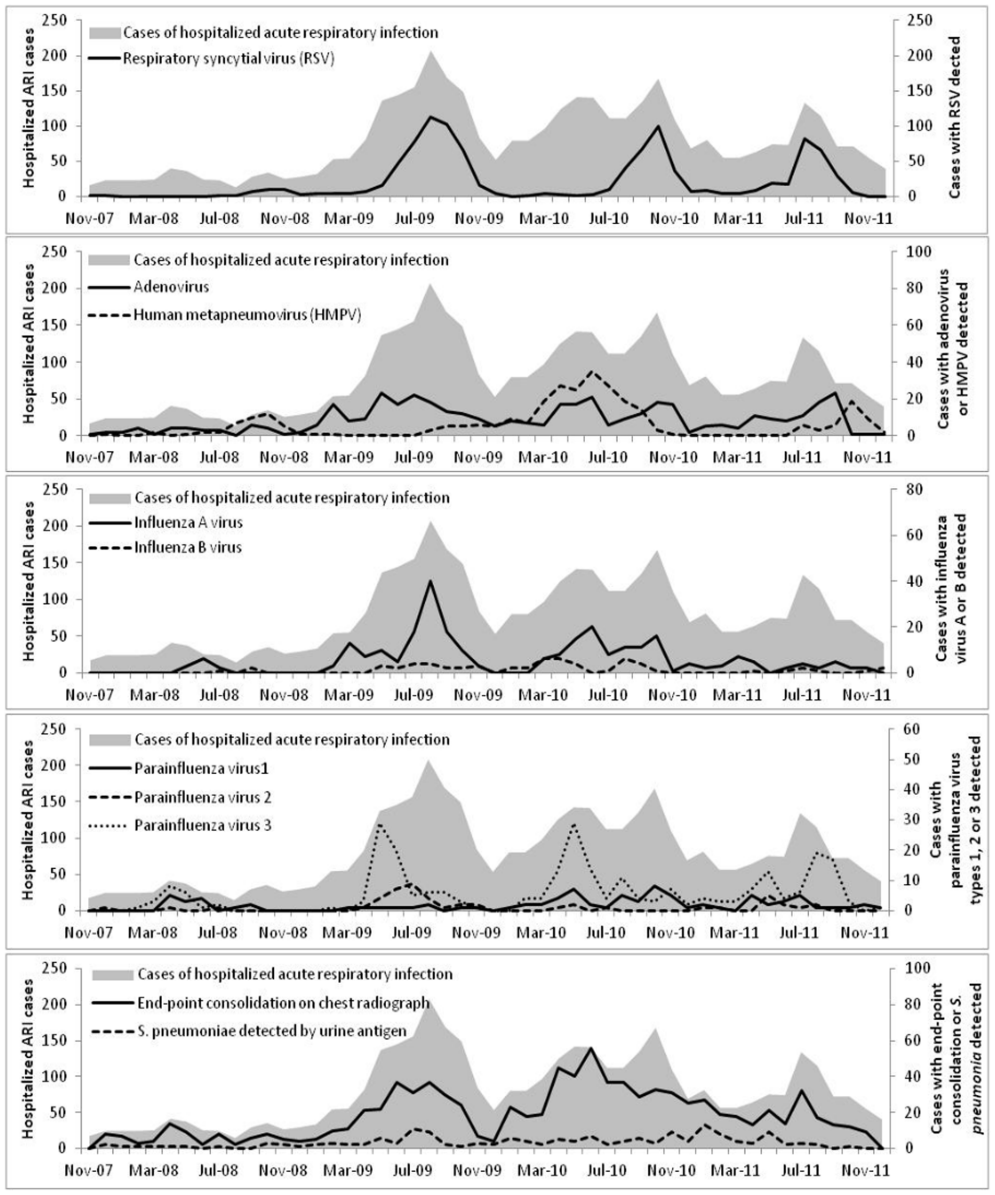
Seasonality of respiratory pathogens and chest radiograph findings. Cases of hospitalized acute respiratory infection (left axis) and number of case-patients with positive results for viral pathogens, *Streptococcus pneumoniae* urine antigen or with endpoint consolidation on chest radiograph (right axis, with varied scales), by month.

The seasonality of hospitalized ARI, viral pathogens, end-point consolidation and *S. pneumoniae* detected by urine antigen (in adult case-patients) is presented in Figure 3, which has varying axes (and therefore does not reflect relative burden of the pathogens). Peaks in the overall number of hospitalized ARI cases were generally observed in the second and third quarters. RSV cases demonstrated a consistent seasonality (peaks in July-November) that closely mirrored the peaks in overall hospitalized ARI cases. Increases in adenovirus cases were also noted during the annual peaks in overall cases, although the pattern was not as clearly defined as that of RSV. Monthly cases of human metapneumovirus were highest in mid-2010, and cases of influenza A virus peaked in mid-2009. Parainfluenza virus type 3, while affecting a relatively small number of cases, had a clear seasonal pattern with peaks in March to July. No seasonality was noted in the monthly cases of pneumococcal infections detected by urine antigen. The number of cases with end-point consolidation on CXR generally increased as the number of overall hospitalized ARI cases increased, however the monthly patterns varied from year to year.

Between 2008 and 2011 the overall incidence of hospitalized ARI in the defined catchment area ranged from 106 to 156 cases per 100,000 people (Table 4). The year with the highest observed incidence (156) was 2009, and there were no clear trends over time. The incidence rate among children <1 year old was 1,703 cases per 100,000– more than five times greater than that of any other age group. Among adults ≥65 years old the incidence was 292 per 100,000. The lowest incidence rate was observed among people between the ages of 5 and 49 years old (34 to 35 cases per 100,000). Although the observed incidence in Santa Rosa was notably higher than that of Quetzaltenango in 2009 (193 [95% CI 177, 211] versus 128 [95% CI 116, 141] per 100,000), the rates across the sites were more similar in 2010 and 2011. Adjusting for reported healthcare-seeking behaviors in the catchment area, the overall estimated rate for hospitalized acute respiratory infection was 263 cases per 100,000 during the study period.

**Table 4:** Observed incidence of hospitalized acute respiratory infections (ARI) in catchment areas by age group, site and incidence adjusted for healthcare utilization patterns, 2008–2011.

	2008		2009		2010		2011		Overall	
	n	Incidence per 100,000 (95% CI)	n	Incidence per 100,000 (95% CI)	n	Incidence per 100,000 (95% CI)	n	Incidence per 100,000 (95% CI)	n	Incidence per 100,000 (95% CI)
**Overall**	260	106 (93, 119)	908	156 (146, 167)	780	125 (116, 134)	730	114 (106, 123)	2678	128 (123, 133)
**Age group**										
<1 year	91	1188 (962, 1452)	418	2306 (2093, 2536)	306	1591 (1420, 1777)	282	1454 (1292, 1631)	1097	1703 (1605, 1806)
1–4 years	76	259 (206, 323)	195	280 (243, 321)	181	244 (211, 282)	119	159 (132, 189)	571	230 (212, 250)
5–14 years	17	25 (15, 40)	70	46 (36, 57)	61	37 (29, 48)	46	28 (21, 37)	194	35 (31, 41)
15–49 years	22	20 (13, 30)	115	43 (36, 51)	83	29 (23, 35)	104	35 (29, 42)	324	34 (30, 37)
50–64 years	23	119 (77, 176)	39	87 (63, 118)	64	134 (104, 170)	61	125 (96, 159)	187	116 (101, 134)
≥65 years	31	234 (164, 328)	71	247 (194, 310)	85	275 (221, 338)	118	372 (309, 444)	305	292 (260, 326)
**Site**										
Santa Rosa	260	106 (93, 119)	485	193 (177, 211)	321	125 (112, 140)	335	128 (115, 143)	1401	138 (112, 125)
Quetzaltenango*	–	–	423	128 (116, 141)	459	124 (113, 136)	395	105 (95, 115)	1277	119 (112, 125)
**Overall hospitalized ARI adjusted for healthcare utilization** †	630	256	1895	326	1520	243	1452	227	5496	263

Surveillance in Quetzaltenango began in February, 2009.

Observed incidences were divided by the following proportions to account for hospitalized ARI cases estimated to have been missed by the surveillance system: Santa Rosa <5 years: 0.33; Santa Rosa ≥5 years: 0.75; Quetzaltenango <5 years: 0.75; Quetzaltenango ≥5 years: 0.50; confidence intervals were not calculated for adjusted rates.

Abbreviations: CI = confidence interval.

## Discussion

These surveillance data demonstrate the importance of hospitalized ARI as a public health problem in Guatemala, and also highlight the dynamic and complex nature of ARI. We found that, at a minimum, 128 of every 100,000 persons and nearly 2 of every 100 children <1 year old are hospitalized for acute respiratory infection each year in the surveillance catchment areas. Nearly 6% of patients hospitalized with ARI died during that admission; and while post-discharge follow-up was limited, an additional 2.7% of those case-patients with available follow-up data had died within 7 days of discharge. The case-fatality proportion was higher among adults than among children; however, given the high incidence of hospitalized ARI among young children, a proportion of 3-5% of cases dying represents a large burden of death due to ARI. Taking into account the reported healthcare utilization patterns, the true burden of hospitalized ARI may be more than twice the incidence observed in this study, as suggested by the estimated adjusted incidence. While all age groups were affected, the greatest burden of hospitalized ARI was observed among young children – a pattern consistent with other published literature [17]. The relatively high burden among older adults (>65 years) is also similar to previously described age patterns of ARI and pneumonia [18]–[20].

The relative contribution of the respiratory pathogens that were studied varied by age group, season and study year. Among viral pathogens, RSV was the most commonly detected, affecting more than a quarter of all case-patients and more than a third of those <1 year old. The predominance of RSV among children with severe respiratory infections and the clear seasonal pattern observed are consistent with studies from a wide variety of settings [21], [22], including data reported from this same surveillance system in Guatemala that focused on RSV in young children and infants [23]. The burden of RSV among adults is less understood, particularly in developing countries. In high-income settings RSV has been recognized as a cause of respiratory disease among certain high risk adult groups [24], and a recent study in Kenya reported a significant association between RSV and hospitalized ARI among older children and adults [25]. We detected RSV among case-patients of all ages, although the relative proportion affected was much higher among young children. Adenovirus, human metapneumovirus, influenza A virus, and parainfluenza virus type 3 were found in 7–11% of case-patients. Other studies in low and middle income countries have also reported these viruses to be relatively common among persons hospitalized with ARI [25]–[32]. The results of virologic testing on NP/OP swabs must be interpreted with caution, however, since some viruses may be present in the nasopharynges of healthy people [25], [26], [33]–[36]. While all of the viruses tested for in the surveillance system are known to cause respiratory disease and pneumonia, the strength of association between detection on NP/OP swabs and illness may vary considerably. Detection of adenovirus, in particular, has not been found to be reliably associated with respiratory illness [37], [38]. Further exploration of viruses present in healthy people from the study area is needed to fully understand the role of viral respiratory pathogens in this context.

The frequency of detection of certain viruses such as human metapneumovirus and influenza A virus in this study varied substantially from year to year. In 2009, the study year with the largest number of hospitalized ARI cases, Guatemala was affected by the emergence of influenza A virus strain (H1N1)pdm09 [39]. Yet even at the peak of the outbreak, the monthly number of RSV infections among case-patients was higher than that of influenza A virus, highlighting the importance of diagnostic testing in the context of outbreaks in order to guide prevention efforts.

Among the bacterial pathogens measured, *S. pneumoniae* was the most frequently detected –15% of adults were found to have evidence of pneumococcal infection by urine antigen assay. This important diagnostic tool for *S. pneumoniae*, however, cannot be used in young children (who suffer the greatest burden of pneumococcal disease) because nasopharyngeal colonization is very common and may lead to false positive urine antigen results [40]. In adults, urine antigen assays for *S. pneumoniae* are considered highly specific, yet the sensitivity has been estimated to be only 50–75% [41]. Therefore the true burden of *S. pneumoniae* is likely much higher than what was observed in this study. NP/OP swabs can be used to aid in the diagnosis of certain bacterial respiratory pathogens that do not tend to colonize the nasopharynx such as *M. pneumoniae* and *C. pneumoniae*; however, the contribution of those bacteria to the hospitalized ARI cases in Guatemala was minimal. Blood culture is highly specific for bacterial etiologies, yet the sensitivity is poor even in optimal conditions [12], [42]; high rates of contamination and frequent prior antibiotic use in this context likely further reduced the utility of blood culture to determine the etiology of hospitalized ARI. *S. aureus* was the most common bacterial pathogen detected by blood culture; however, given the rates of contamination observed it is possible that some of those cases may represent contamination rather than infection. Standardized CXR interpretation according to WHO guidelines is an important epidemiologic tool for estimating the burden of probable bacterial respiratory infections among children [10], [12], although experience with expanding these techniques to adult radiographs is limited [18]. End-point consolidation, which was found in 40% of case-patients, is suggestive of a bacterial etiology but cannot distinguish between bacterial pathogens. However, other studies in Guatemala and other Latin American countries have found *S. pneumoniae* to be the leading bacterial cause of pneumonia in children [43], [44] and adults [2], [45].

Evidence of infection with more than one pathogen was common, with nearly one in ten case-patients testing positive for multiple viruses and 29% or more of those with a bacterial infection also testing positive for at least one virus. Viral-bacterial co-infections have been described in up to 45% of cases of pediatric community-acquired pneumonia, with *S. pneumoniae* and a respiratory virus being the most typical combination [46]. Although data are limited on the clinical significance of multiple concurrent viruses and bacterial-viral co-infections, there is some evidence that such infections may be more severe and associated with poorer outcomes compared with single pathogen infections [46]–[48].

The findings of this study are subject to several limitations. First, the case definition for hospitalized ARI is based on signs/symptoms of respiratory disease and signs of acute infection; this differs from other case definitions in the field of respiratory infections (such as severe acute respiratory illness or pneumonia as defined in the Integrated Management of Childhood Illness guidelines [49]) and therefore the results are not directly comparable to studies using those definitions. The surveillance is hospital-based, so the findings cannot be extrapolated to persons with ARI that seek care elsewhere or do not seek medical care. Adjusted estimates of incidence were based on baseline healthcare utilization surveys conducted in the catchment area of each site and attempted to account for patients that may have been admitted with ARI to hospitals other than the surveillance sites; however, they do not account for people who were not hospitalized or did not seek care for their respiratory illness. It is also possible that healthcare utilization may have changed over time, which would affect the observed and adjusted incidences. As noted above, the determination of the etiology of ARI is limited by available diagnostic tools, including potentially poor positive predictive value of viral testing of NP/OP swabts and the relative insensitivity of blood culture and urine antigen testing for bacterial pathogens.
